# Effect of Subthalamic Stimulation and Electrode Implantation in the Striatal Microenvironment in a Parkinson’s Disease Rat Model

**DOI:** 10.3390/ijms232012116

**Published:** 2022-10-11

**Authors:** Ana Carolina Pinheiro Campos, Raquel Chacon Ruiz Martinez, Aline Vivian Vatti Auada, Ivo Lebrun, Erich Talamoni Fonoff, Clement Hamani, Rosana Lima Pagano

**Affiliations:** 1 Laboratory of Neuroscience, Hospital Sírio-Libanês, São Paulo 01308-901, Brazil; 2 Medical Investigation Laboratory/23, Institute of Psychiatry, School of Medicine São Paulo University, São Paulo 05403-903, Brazil; 3 Biochemistry and Biophysics Laboratory, Butantan Institute, São Paulo 05508-040, Brazil; 4 Department of Neurology, University São Paulo, São Paulo 05508-900, Brazil; 5 Brain Sciences Program, Sunnybrook Research Institute, Toronto, ON M4N 3M5, Canada

**Keywords:** Parkinson’s disease, deep brain stimulation, subthalamic stimulation, neuroinflammation, astrocytes, glutamate excitotoxicity

## Abstract

Deep brain stimulation (DBS) of the subthalamic nucleus (STN) is considered the gold-standard treatment for PD; however, underlying therapeutic mechanisms need to be comprehensively elucidated, especially in relation to glial cells. We aimed to understand the effects of STN-microlesions and STN-DBS on striatal glial cells, inflammation, and extracellular glutamate/GABAergic concentration in a 6-hydroxydopamine (6-OHDA)-induced PD rat model. Rats with unilateral striatal 6-OHDA and electrodes implanted in the STN were divided into two groups: DBS OFF and DBS ON (5 days/2 h/day). Saline and 6-OHDA animals were used as control. Akinesia, striatal reactivity for astrocytes, microglia, and inflammasome, and expression of cytokines, cell signaling, and excitatory amino acid transporter (EAAT)-2 were examined. Moreover, striatal microdialysis was performed to evaluate glutamate and GABA concentrations. The PD rat model exhibited akinesia, increased inflammation, glutamate release, and decreased glutamatergic clearance in the striatum. STN-DBS (DBS ON) completely abolished akinesia. Both STN-microlesion and STN-DBS decreased striatal cytokine expression and the relative concentration of extracellular glutamate. However, STN-DBS inhibited morphological changes in astrocytes, decreased inflammasome reactivity, and increased EAAT2 expression in the striatum. Collectively, these findings suggest that the beneficial effects of DBS are mediated by a combination of stimulation and local microlesions, both involving the inhibition of glial cell activation, neuroinflammation, and glutamate excitotoxicity.

## 1. Introduction

Parkinson’s disease (PD) is a neurodegenerative disorder characterized by progressive loss of dopaminergic neurons in the nigrostriatal pathway [1]. Beyond dopamine deficits, neuroinflammation is a pivotal phenomenon of PD [2,3]. From this perspective, activated microglia in post-mortem tissue or observed by positron emission tomography, as well as an increased expression of pro-inflammatory cytokines, have been detected in the cerebrospinal fluid (CSF) and substantia nigra (SN) of patients with PD [4,5,6]. Considering that microglia are primary resident immune cells of the central nervous system, their activation is considered pivotal for protecting the brain parenchyma [7]. However, with extensive neuronal loss, constant microglial activation alters their phenotypic polarization to classically activated microglia (M1), which releases pro-inflammatory cytokines (i.e., tumor necrosis factor (TNF)-α, interleukin (IL)-1β and chemokine (C-X3-C motif) ligand 1 (CX3CL1)), consistent with auto-sustained local inflammation [8,9,10,11]. Furthermore, astrocytes, the most abundant cells of the central nervous system [12], can be classically activated (A1 phenotype) by damage-associated molecular patterns released by neuronal death, as well as by M1 microglia releasing further pro-inflammatory mediators [13]. In addition to their role in inflammation, astrocytes can mediate a series of important functions in the central nervous system. These cells are responsible for regulating cerebral blood flow and the concentration of ions, metabolites, and neurotransmitters that actively participate in synapse pruning [14,15,16,17,18]. One major role of astrocytes involves glutamate uptake by excitatory amino acid transporter 2 (EAAT2; for rodents or GLT-1 for humans), representing approximately 90% of glutamatergic clearance in the central nervous system [19,20,21,22]. However, once classically activated, astrocytes lose their ability to prune synapses and control glutamate-mediated excitotoxicity and oxidative stress [23,24]. Therefore, controlling inflammation in PD is pivotal for inhibiting aberrant synaptic signaling that leads to neuronal excitotoxicity and death, factors implicated in the pathogenesis and progression of PD.

Regarding available treatments for PD, dopamine replacement using levodopa remains the standard drug therapy [25]; however, this treatment can eventually result in side effects such as dyskinesia, which deteriorates the quality of life of individuals with PD [26,27]. In this phase, deep brain stimulation (DBS) of the subthalamic nucleus is considered the gold-standard treatment for improving motor symptoms of PD [28,29,30,31,32,33,34]. However, the mechanisms underlying DBS are still under investigation. Hamani and coworkers have summarized the main mechanisms through which subthalamic stimulation may improve PD symptoms [35]. However, it is crucial to consider that preclinical models are the most common source of molecular-based mechanisms for numerous neurological diseases and treatment strategies. Therefore, it is pivotal to understand the therapeutic effect of the implantation-induced lesion, the so-called “insertion effect”, in a preclinical setting and determine the combined effect of electrode implantation and stimulation. Several of the proposed DBS mechanisms include neuronal-based local mechanisms. In contrast, Fenoy et al. have postulated that astrocytes might be crucial in DBS neuroplasticity mechanisms by releasing gliotransmitters, modulating neuronal circuitry and synapsis, increasing neurotrophic factors, and inhibiting inflammation [16].

Herein, we aimed to identify changes induced by the insertion effect in the STN and STN stimulation in the striatum of animals subjected to 6-hydroxydopamine (6-OHDA)-induced PD, focusing on the inflammatory response and glutamate excitotoxicity.

## 2. Results

### 2.1. STN-DBS Alleviates Motor Impairment and Modulates Astrocytes Morphology in the Striatum

**Figure 1:**
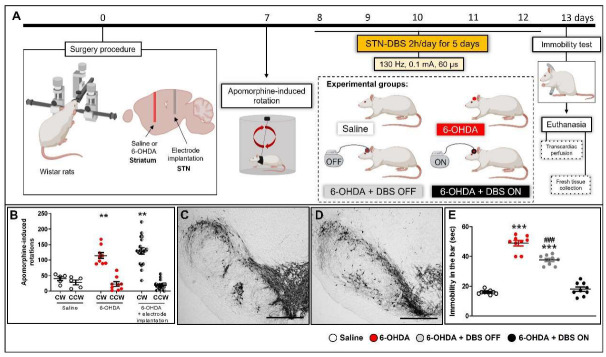
Experimental design (**A**)**.** Apomorphine-induced rotation (**B**) was performed 7 days after the surgical procedure on saline (*n* = 5), 6-OHDA (*n* = 9), and implanted 6-OHDA animals (*n* = 19, divided into DBS OFF and DBS ON animals). Data values represent the individual values and mean ± SEM. Two-way ANOVA followed by Tukey’s multiple comparison post-hoc. ** *p* < 0.01 vs. control saline group. Representative photomicrographs of tyrosine hydroxylase immunoreactivity in the striatum of saline (**C**) and 6-OHDA (**D**) groups. The immobility test (**E**) was performed 13 days after the surgical procedure (24 h after the last DBS session) on saline (*n* = 10), 6-OHDA (*n* = 10), 6-OHDA + DBS OFF (*n* = 10) and 6-OHDA + DBS ON (*n* = 10). Data values represent the individual values and mean ± SEM. One-way ANOVA followed by Tukey’s multiple comparison post-hoc. *** *p* < 0.001 vs. control saline group; ^###^ *p* < 0.001 when compared to 6-OHDA group. Immediately after the immobility test, some animals were euthanized by transcardiac perfusion, while others were euthanized by decapitation for fresh brain tissue collection. Scale bar: 100 µm. 6-OHDA: 6-hydroxydopamine; DBS: deep brain stimulation; CW: clockwise; CCW: counter-clockwise; STN: subthalamic nucleus. Figure constructed by the author using BioRender.com (accessed on 20 July 2022).

Animals administered striatal 6-OHDA showed increased asymmetric rotations (F_(2,48)_ = 38.37, *p* < 0.0001; Figure 1B) and decreased TH immunoreactivity (IR) in the SN (Figure 1D) when compared with saline-injected control rats (Figure 1B,C). Similar to our previous findings [36], electrode implantation per se (6-OHDA + DBS OFF group) failed to prevent apomorphine-induced asymmetric rotational behavior (Figure 1B). In contrast, electrode implantation in the STN induced an evident microlesion effect on alternative motor behavior, as previously demonstrated by our group [36]. Compared with saline-injected rats, 6-OHDA animals exhibited increased immobility time on the bar (*p* < 0.0001). Implanted but unstimulated animals (with STN-microlesions) showed a partial and discreet decrease in 6-OHDA-induced immobility time (*p* < 0.0001, when compared with saline and 6-OHDA-injected animals), whereas stimulated animals (STN-DBS) showed complete inhibition of the akinesia phenomenon when compared with controls (F_(3,32)_ = 147.6, *p* < 0.0001; Figure 1E).

**Figure 2:**
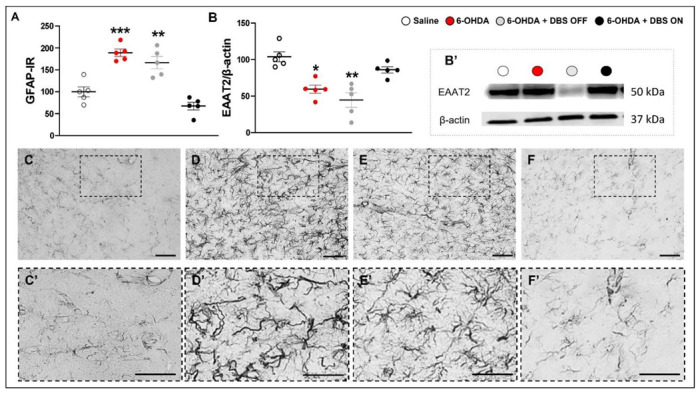
Effect of STN-microlesion or STN-DBS treatment on striatal astrocytes and EAAT2. Striatal immunoreactivity (IR) of GFAP (**A**) and EAAT2 expression (**B**) were evaluated in saline, 6-OHDA, 6-OHDA + DBS OFF, and 6-OHDA + DBS ON groups. *n* = 5 per group. Data values represent the individual values and mean ± SEM. One-way ANOVA followed by Tukey’s multiple comparison post-hoc. * *p* < 0.05; ** *p* < 0.01; *** p < 0.001 vs. saline group. Representative western blot bands (**B**′). Representative photomicrographs of immunoreactivities for GFAP (astrocytes, **C**–**F**) on the striatum of saline (**C**,**C**′), 6-OHDA (**D**,**D**′), 6-OHDA + DBS OFF (**E**,**E**′), and 6-OHDA + DBS ON (**F**,**F**′) rats. Scale bar: 100 µm. 6-OHDA: 6-hydroxydopamine; DBS: deep brain stimulation; EAAT: excitatory amino acid transporter.

We observed a marked increase in GFAP-IR (F_(3,15)_ = 27.05, *p* < 0.0001; Figure 2A) accompanied by the presence of the NLPR3 inflammasome marker (Figure S1) and decrease in EAAT-2 expression (F_(3,15)_ = 10.79, *p* = 0.001; Figure 2B) in 6-OHDA and 6-OHDA + DBS OFF when compared with saline rats, an effect that was mitigated in 6-OHDA + DBS ON rats.

### 2.2. Subthalamic Microlesion and Stimulation Inhibit 6-OHDA-Induced Striatal Cytokines while Only STN-DBS Regulates Glutamate Reuptake and Neuronal Autophagy without Interfering with Microglial Immunoreactivity

**Figure 3:**
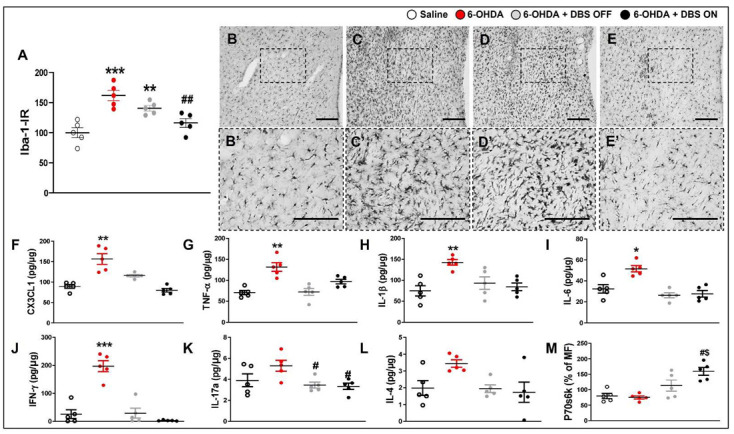
Effect of STN-microlesion or STN-DBS treatment on striatal microglia and inflammatory mediators. Striatal immunoreactivity (IR) of Iba-1 (**A**) and representative photomicrographs of immunoreactivities for GFAP (astrocytes, **B**–**E**) on the striatum of saline (**B**,**B**′), 6-OHDA (**C**,**C**′), 6-OHDA + DBS OFF (**D**,**D**′), and 6-OHDA + DBS ON (**E**,**E**′) rats. Scale bar: 100 µm. Striatal expression levels of CX3CL1 (**F**), TNF-α (**G**), IL-1β (**H**), IL-6 (**I**), IFN-y (**J**), IL-17A (**K**), IL-4 (**L**), and p70s6k (**M**) were evaluated in saline, 6-OHDA, 6-OHDA + DBS OFF, and 6-OHDA + DBS ON groups. *n* = 5 per group. Data values represent the individual values and mean ± SEM. One-way ANOVA followed by Tukey’s multiple comparison post-hoc. * *p* < 0.05; ** *p* < 0.01; *** *p* < 0.001 vs. saline group. ^#^ *p* < 0.05; ^##^ *p* < 0.01 vs. 6-OHDA group; ^$^ *p* < 0.05 vs. 6-OHDA + DBS OFF group. 6-OHDA: 6-hydroxydopamine; CX3CL1: chemokine (C-X3-C motif) ligand 1; DBS: deep brain stimulation; IFN-γ: interferon-γ; IL: interleukin; MF: median fluorescence; TNF-α: tumor necrosis factor-α.

To further investigate the effect of STN-DBS on neuroinflammation and the control of intracellular signaling, we evaluated the striatal immunoreactivity of microglia, the expression of cytokines, and cell signaling markers. Compared with saline rats, 6-OHDA rats exhibited increased immunoreactivity of Iba-1 (F_(3,15)_ = 13.70, *p* < 0.0001; Figure 3B), and increased expression of CX3CL1 (F_(3,15)_ = 12.50, *p* = 0,00005; Figure 3F), TNF-α (F_(3,15)_ = 8.534, *p* = 0.0026; Figure 3G), IL-1β (1-w-ANOVA; F_(3,15)_ = 6.822, *p* = 0.0040; Figure 3H), IL-6 (F_(3,15)_ = 9.193, *p* = 0.0016; Figure 3I), and IFN-γ (F_(3,15)_ = 20.46, *p* < 0.0001; Figure 3J). IL-17A expression was inhibited in 6-OHDA + DBS OFF and 6-OHDA + DBS ON rats (F_(3,15)_ = 5.504, *p* = 0.0130; Figure 3K). Six-OHDA + DBS OFF and 6-OHDA + DBS ON failed to show a significant decrease in Iba-1-IR when compared to saline animals; however, stimulated animals showed a discrete increase in Iba-1-IR when compared to 6-OHDA rats (*p* = 0.0023; Figure 3A). Both treatments counteracted the increased expression of CX3CL1, TNF-α, IL-1β, IL-6, and IFN-γ (Figure 3). We detected no difference in striatal IL-4 expression between experimental groups (F_(3,15)_ = 2.294, *p* = 0.1299; Figure 3L). Regarding the expression of the neuronal autophagy marker p70s6k, only the 6-OHDA + DBS ON group showed increased expression when compared with other groups (F_(3,15)_ = 6.32, *p* = 0.0081; Figure 3M). The cytokine IL-10 and intracellular markers Akt, CREB, Erk, JNK, p65, p38, STAT3, and STAT5A were analyzed; however, expression levels were either undetectable or detectable under low quantification limits in striatal tissue samples from different experimental groups.

### 2.3. Subthalamic Microlesion and Stimulation Inhibit the Relative Concentration of 6-OHDA-Induced Glutamate

**Figure 4:**
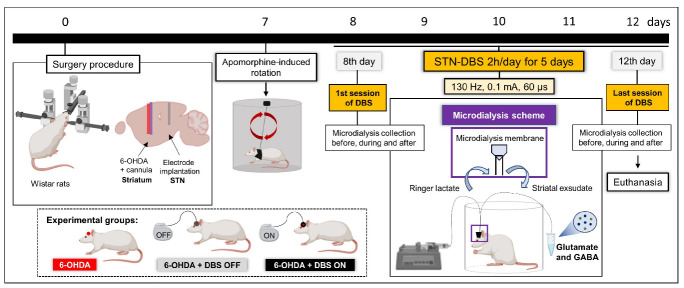
Experimental design striatal microdialysis in animals. 6-OHDA animals with or without electrode implantation underwent implantation of a guided cannula in the left striatum. Striatal exudate was collected before and after the first and fifth DBS session, corresponding to days 8 and 12 after striatal neurotoxin injection. 6-OHDA and 6-OHDA + DBS OFF were collected for 2 h under the same conditions as stimulated animals. 6-OHDA: 6-hydroxydopamine; DBS: deep brain stimulation; PD: Parkinson’s disease; STN: subthalamic nucleus. Figure constructed by the author using BioRender.com (2022).

Considering that glutamatergic-induced excitotoxicity is critical for the progression of neurodegeneration in PD [37,38], we evaluated the relative concentration of striatal glutamate and GABA in hemiparkinsonian rats without electrodes, with STN-microlesions, and STN-DBS, as described above (Figure 4).

**Figure 5:**
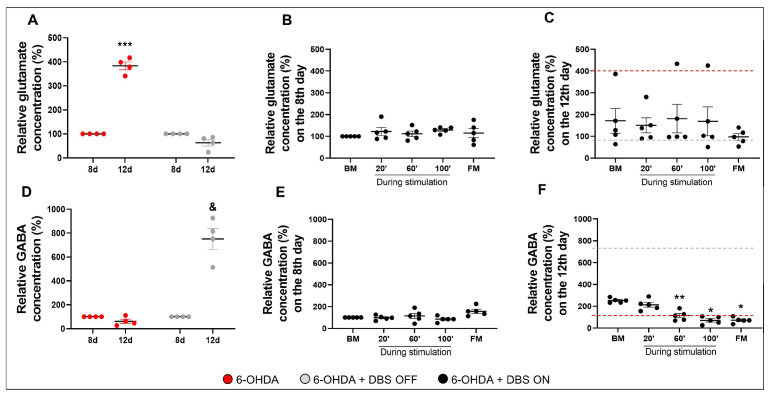
Effect of STN-microlesion or STN-DBS treatment on striatal glutamate and GABA relative concentration. Animals were habituated for 90 min before striatal microdialysis collection on days 8 and 12 after the surgical procedure in 6-OHDA (*n* = 4), 6-OHDA + DBS OFF (*n* = 4), and 6-OHDA + DBS ON (*n* = 5) animals. Mean relative striatal glutamate) concentration in 6-OHDA and 6-OHDA + DBS OFF groups (**A**) and 6-OHDA + DBS ON rats before, during, and after the first DBS session (day 8 after the lesion, **B**) and after the fifth and last session of DBS (day 12 after the lesion, **C**). Mean relative striatal GABA concentration in 6-OHDA and 6-OHDA + DBS OFF groups (**D**) and 6-OHDA + DBS ON rats before, during, and after the first DBS session (day 8 after the lesion, **E**) and after the fifth and last session of DBS (day 12 after the lesion, **F**). Data values represent the individual values and mean ± standard error of the mean (SEM). Two-way ANOVA. *** *p* < 0.001 vs. 6-OHDA on day 8 after the nigrostriatal lesion. ^&^ *p* < 0.05 vs. 6-OHDA + DBS OFF rats on day 8 after the nigrostriatal lesion. Data values represent the individual values and mean ± SEM. All data regarding the glutamate (**C**) and GABA (**F**) before, during and after the 5th and last session of DBS performed 12 days after the nigrostriatal lesion was normalized by the basal measurement of the 8th day. One-way repeated measures ANOVA. * *p* < 0.05; ** *p* < 0.01 vs. BM after day 12 of nigrostriatal lesion. The red line indicates the mean relative concentration of 6-OHDA animals; the gray line represents the mean relative concentration of 6-OHDA + DBS OFF animals. 6-OHDA: 6-hydroxydopamine; BM: basal measurement; DBS: deep brain stimulation; FM: final measurement; GABA: γ-aminobutyric acid; PD: Parkinson’s disease.

In the 6-OHDA group, we observed an approximately 200% increase in the relative glutamate concentration between days 8 and 12 after the nigrostriatal lesion; STN-microlesions in 6-OHDA + DBS OFF animals could suppress increased striatal glutamate release (F_(1,8)_ = 98.34, *p* < 0.0001, followed by Tukey’s post hoc test; Figure 5A). Interestingly, subthalamic stimulation (6-OHDA + DBS ON group) did not induce any significant differences in striatal glutamatergic release during or after the first DBS session (day 8 following 6-OHDA administration) (F_(1,19)_ = 0.4, *p* = 0.6; Figure 5B) and after the fifth DBS session (day 12 following 6-OHDA administration) (F_(1,19)_ = 1.12, *p* = 0.38; Figure 5C) when compared with the basal measurement. Considering that disrupted glutamate and GABA systems have been associated with the progression of PD and synaptic instability [39], we investigated the relative concentration of striatal GABA in different experimental groups. 6-OHDA animals did not show any difference between days 8 and 12 after induction of the nigrostriatal lesion, whereas 6-OHDA + DBS OFF animals showed a significant increase in the striatal relative concentration of GABA (F_(1,2)_ = 38.21, *p* = 0.025; Figure 5D). Additionally, 6-OHDA + DBS ON animals displayed no significant differences during and after the first DBS session (F_(1,12)_ = 2.5, *p* = 0.1; Figure 5E). However, STN-DBS decreased striatal GABA release 60 min after the last DBS session. This inhibition was maintained until the final analysis, 60 min after stimulation offset (F_(1,19)_ = 19.34, *p* = 0.01; Figure 5F).

## 3. Discussion

STN-DBS has been widely used to treat patients with PD who developed undesirable side effects after dopaminergic treatment. This therapy is associated with excellent outcomes in terms of motor and non-motor symptoms of PD [28,29,30,31,32,33,34]. Glia cell modulation has been implicated in mediating the underlying mechanism of DBS but warrants further clarification. In the present study, we aimed to elucidate the striatal anti-inflammatory effects of subthalamic stimulation in a rodent PD model. Herein, we showed that our PD model, validated by increased asymmetric rotational behavior and inhibition of TH-IR in the SN, induced motor impairment and morphological changes (i.e., hypertrophic and hyperplasia phenomena) in astrocytes and microglia, activated the inflammasome component, increased pro-inflammatory cytokine expression, and inhibited the astrocytic-mediated control of excitotoxicity, increasing the release of striatal glutamate. In addition, animals implanted with electrodes, which remained unstimulated, showed partial improvement in motor symptoms, inhibition of pro-inflammatory cytokines, and prevention of glutamate release associated with 6-OHDA-induced lesions. Stimulated animals exhibited evident motor improvement and inhibited striatal cytokine and glutamate levels. In addition to the microlesion-induced effect, STN-DBS inhibited glial cells in the striatum (decreased number of cells and morphology), inflammation, increased expression of EAAT2 (astrocytic amino acid transporter), and elevated GABA levels in the last DBS session (5th session).

In preclinical settings, assessing the insertional effect (electrode implantation but no stimulation) is critical, especially considering the proportional size of the electrode when compared with rodent STN and the fact that it may induce considerable tissue disruption, as demonstrated by Nissl staining [36]. Furthermore, it has been shown that the insertional effect itself may induce motor improvement even prior to initiating stimulation in individuals [40,41]. Indeed, STN-microlesions may present therapeutic responses similar to those observed in thalamotomies [35,42,43]. However, unlike therapeutic DBS, stereotactic ablative procedures are irreversible and may induce debilitating and permanent adverse effects [44,45]. Therefore, clarifying clinical and molecular differences and similarities between STN-microlesions and DBS remains essential for improving therapeutics in PD. Initially, we evaluated the effect of STN-microlesions or STN-DBS on akinesia, a well-established behavior in the 6-OHDA model [46,47]. As previously reported by our research group [36], striatal neurotoxins can cause forelimb akinesia, which is significantly improved by STN-microlesions and completely reversed following STN-DBS, without interfering with the decrease in dopaminergic neurons and fibers in the SN. A similar effect has also been reported in patients with PD, lasting 7–14 days [40,41,43]. Nevertheless, therapeutic lesions induced by radiofrequency or MR-guided focused ultrasound have a role in clinical practice [48,49,50].

Inflammation is an important hallmark of PD [51]. Glial cell activation in the striatum has been previously demonstrated [2]; however, it is important to consider that although induction of the PD model is triggered by striatal 6-OHDA inoculation, no focal neuronal death was detected (Supplemental Figure S1), suggesting that the inflammation observed in this nucleus may be attributed to nigral neurodegeneration. In these animals, astrocytes and microglia were hypertrophic and hyperplastic, which are changes often observed in classic activation states [52]. To the best of our knowledge, this is the first study to demonstrate inflammasome activation in the striatum of a PD model. Nevertheless, inflammasome has been shown to be present in both astrocytes and microglia in the presence of sterile neuroinflammation [53]. Interestingly, the increased striatal inflammatory response is accompanied by inhibited EAAT2 expression, as well as a progressive increase in the relative glutamate concentration, corroborating the finding that classically activated astrocytes fail to reuptake glutamate [13]. Furthermore, Chung et al. have shown a decrease in striatal EAAT2 expression in a rat PD model; however, this decrease was not observed in the SN or neuronal glutamatergic transporters [54]. Likewise, it has been shown that extracellular glutamate results in aberrant synaptic signaling and is associated with glial reaction and neuroinflammation [37]. Moreover, inefficient clearance may lead to a deleterious increase in striatal glutamate, inducing excitotoxicity and contributing to progressive inflammation and neurodegeneration [55,56]. This emphasizes the importance of glutamatergic clearance in the striatum and the pivotal role of astrocytes in glutamate reuptake, thus mitigating excitotoxicity in PD conditions.

Interestingly, we observed that non-stimulated, unimplanted hemiparkinsonian animals showed increased striatal levels of pro-inflammatory cytokines when compared with those detected in control animals. Furthermore, electrode implantation per se (STN-microlesion) suppressed cytokine release within the striatum without interfering with exacerbated glial cell activation (observed by the number and morphology of astrocytes and microglia), inflammasome activation, or a decrease in EAAT2 expression. Conversely, subthalamic stimulation modulated the morphological pattern and cell number of astrocytes and microglia, suggesting a more pronounced control of striatal inflammation, which was corroborated by the decreased expression of pro-inflammatory cytokines and increased EAAT2. A similar DBS-mediated anti-inflammatory effect has been reported in an epilepsy model with an associated decrease in apoptosis [57]. Indeed, it has been shown that increased EAAT2 expression plays a protective role in cultured neurons [58]. In addition, optimal clearance of glutamate is possibly critical for preventing the exacerbated activation of NMDA receptors and avoiding excitotoxicity [59,60]. Hence, we suggest that STN-DBS exerts a more prominent anti-inflammatory effect than the microlesion itself. Considering a previous observation that astrocytes may be responsible for the neuroplasticity observed after chronic DBS [16], we propose that the cellular pattern observed in our stimulated animals may explain its therapeutic superiority. While the electrode implantation, as described above, induces a transitory therapeutical effect that may be, at least, partially due to the acute inhibition of pro-inflammatory cytokines; the stimulation is able to modulate the morphology and function (glutamate clearance) at the cellular level, which may reflect in a more sustainable therapeutical effect. Nevertheless, it is important to highlight that neuroinflammation and glial activation is a complex mechanism and, rather than a pro- or anti-inflammatory microenvironment in vivo, it is more likely that glial cells present with an array of phenotypes that attenuate or aid the neurodegenerative process altogether [61]. Therefore, it is of most importance to develop further studies regarding the different patterns of glia cells and their modulation in neurodegenerative conditions in an attempt to guide most effective therapeutic strategies. Furthermore, it has been shown that DBS may promote neuroprotection by modulating pivotal mediators of synaptic stimulation and autophagy in Alzheimer’s disease and PD [62]. Furthermore, we found that only stimulated animals exhibited increased p70s6k expression. Given that increased p70s6k expression in astrocytes protects 1-methyl-4-phenyl-1,2,3,6-tetrahydropyridine (MPTP)-inoculated cultured neurons [63], it is reasonable to assume that subthalamic stimulation may modulate astrocytes preferentially to an alternative and neuroprotective phenotype than to a neurotoxic phenotype.

The therapeutic effect of DBS is considered multifactorial, where electrical activity on the target area induces electrical and neurochemical responses. These responses initiate a complex neuroplasticity process, local and network-wide, that may influence neurogenesis and neurodegeneration processes [64]. Therefore, we aim to understand the off-target modulation of pivotal striatal neurotransmitters in PD. We observed a progressive increase in extracellular glutamate, as previously described in the proposed experimental PD model [65], with no apparent interference with GABAergic levels. Considering that we compared rats after the surgical procedure on days 8 and 12, these findings highlight progressive neuronal impairment in the 6-OHDA PD model. Interestingly, STN-microlesions inhibited the abnormal increase in striatal glutamate in rats with nigrostriatal lesions, corroborating the previous findings, revealing that subthalamic lesions and levodopa treatment inhibited the amplitude and frequency of glutamatergic synapses [66], suggesting the importance of controlling glutamatergic release for therapeutic efficacy. Additionally, STN-microlesions increased striatal GABA release in the PD model. It is important to highlight that these animals did not show an increase in striatal EAAT2 expression, and thus glutamate clearance could still be impaired. Therefore, we hypothesized that controlling glutamate clearance in these animals could be attributed to increased GABA induced by STN inhibition. GABA is present in the medium spiny neurons within the striatum and may also be released by dopaminergic efferences to inhibit direct and indirect motor pathways [67,68]. Given that the STN projects directly into the SN [69], its inhibition (mechanically or by DBS) may be reflected in the increased GABA release in the striatum. However, we observed stable glutamate release during and after the first and fifth DBS sessions, which were below the levels observed in PD-induced animals. Interestingly, it has been shown that acute, high-frequency stimulation (as applied in DBS) induces astrocyte-mediated glutamatergic release in vitro [70], as glutamate release was observed after 20 min of STN-DBS in hemiparkinsonian rats [71].

Furthermore, stimulated animals showed stable GABAergic release during and after the first DBS session; however, we observed a two-fold increase in GABAergic release in the basal measurement immediately before the fifth and last DBS sessions. Surprisingly, after 60 min, we detected a significant decrease in GABA release, sustained until the end of stimulation. Twelve days after the nigrostriatal lesion, we observed that STN-microlesions increased the relative GABA concentration by eight-fold in the striatum, which was four times greater than the increase observed at baseline in stimulated animals during the same period. This could suggest that STN-DBS decreases GABAergic release throughout the course of stimulation (from the first to fifth session), possibly due to the reduced necessity to inhibit striatal glutamatergic output because of astrocytic-mediated glutamate clearance or additional neuronal mechanisms of subthalamic stimulation that decrease glutamatergic release into the striatum from the motor cortex and thalamus feedback [69]. Considering the interaction between GABA and glutamate, it has been demonstrated that STN stimulation leads to a reduction in synaptic glutamate release caused by the tonic release of GABA from co-activated striatonigral afferents to the SN pars reticulata, which indicates that STN-DBS sessions modify synaptic transmission, leading to suppression of activity in the output region of the basal ganglia [72].

**Figure 6:**
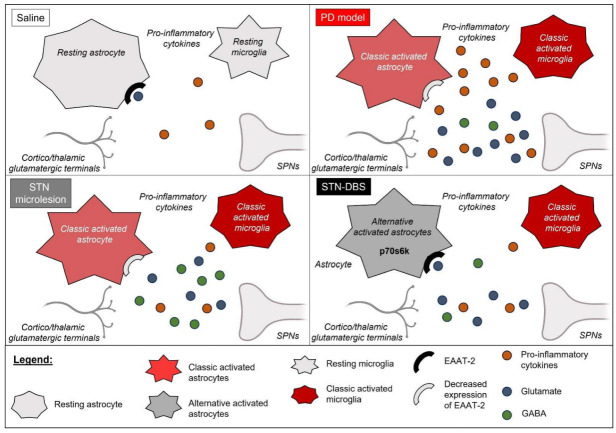
Summary scheme of the main findings. The Parkinson’s disease (PD) rat model exhibits classical astrocyte and microglial activation, as evidenced by the increase in pro-inflammatory cytokines, increase in extracellular glutamate accompanied by the decreased expression of excitatory amino acid transporter (EAAT)-2. The subthalamic (STN) microlesion decreases pro-inflammatory cytokines and inhibits extracellular glutamate without modulating the number or morphology of glial cells and EAAT2 expression. However, STN stimulation can modulate astrocytes, increase EAAT2 expression, and elevate the levels of p70s6k, suggesting that deep brain stimulation (DBS) may modify astrocytic phenotypes from the classic activated/neurotoxic to alternatively activated astrocytes/neuroprotective. Figure constructed by the author using BioRender.com (2022).

Collectively, our data demonstrate that the striatal 6-OHDA-induced PD model induces glial cell activation, along with consequent elevations in pro-inflammatory mediators and glutamatergic levels within the striatum, which can be, at least partially, attributed to the inability of astrocyte-mediated glutamatergic clearance. STN-microlesions, induced by electrode implantation, decrease pro-inflammatory cytokine levels and protect against abnormal glutamate release; however, they fail to modulate the glial profile and the expression of astrocytic amino acid transporters. Conversely, STN-DBS inhibited not only striatal pro-inflammatory mediators but also decreased glial immunoreactivity and inflammasome patterns and increased EAAT2 expression, potentially improving glutamatergic clearance and modulating GABAergic release, thereby suggesting a more pronounced modulation of synaptic dysfunction within the hemiparkinsonian rat striatum (Figure 6).

## 4. Materials and Methods

### 4.1. Experimental Design

Under stereotaxic conditions, rats were randomized to receive either striatal 6-OHDA (PD model) or saline injections (control), as described below. During the same surgical procedure, electrodes were implanted into the left STN of some 6-OHDA animals. The experimental groups were divided as follows: (1) animals injected with striatal saline (control of PD model, *n* = 9); (2) animals injected with striatal 6-OHDA without electrode implantation (control of electrode implantation, *n* = 9); (3) animals injected with striatal 6-OHDA + DBS OFF (only electrode implanted) (*n* = 9); (4) animals injected with striatal 6-OHDA + DBS ON (stimulated) (*n* = 9). Seven days after striatal injection, the animals were evaluated using the apomorphine-induced rotation test (to validate the PD model). The next day, 6-OHDA + DBS ON rats were subjected to five consecutive sessions of DBS (130 Hz, 0.1 mA and 60 µs pulse width) for 2 h daily. Twenty-four hours after the last stimulation session (13 days after the surgical procedure), all experimental groups were evaluated using the immobility test (to evaluate motor symptoms). Thereafter, animals were euthanized by transcardiac perfusion for immunohistochemical staining of tyrosine hydroxylase (a marker of dopaminergic deficit), GFAP (astrocytic marker), Iba-1 (microglial marker), and NLRP3 (inflammasome marker) in the striatum, or underwent decapitation for fresh tissue analyses of striatal expression of inflammatory mediators and EAAT2 (Figure 1A). Moreover, after performing the previously described surgical procedure, another group of animals underwent cannula implantation in the left striatum to evaluate striatal neurotransmitter release. Animals were divided into three groups: 6-OHDA without implantation (*n* = 4), 6- OHDA + DBS OFF (*n* = 4), and 6-OHDA + DBS ON (*n* = 5). These animals were subjected to microdialysis collection before, during, and after the first and last DBS sessions (same protocol as previously described) to evaluate the concentration of glutamate and γ-aminobutyric acid (GABA) released in the left striatum by liquid chromatography (Figure 4).

### 4.2. Animals

A total of 55 male Wistar rats (200–250 g) were used in the present study. Herein, we presented the results from 49 animals, of which 6 animals were excluded due to premature death or improperly placed STN implants. The rats were housed in acrylic boxes (three rats per box) for at least one week before initiating experimental procedures. The animals were maintained in appropriate rooms with a controlled light/dark cycle (12/12 h) and temperature (22 ± 2 °C), with wood shavings and free access to water and rat chow pellets. All animal experiments were conducted in accordance with ARRIVE guidelines (http://www.nc3rs.org.uk/arrive-guidelines, accessed on 5 July 2022). The protocols used during the execution of this project were approved by the Ethics Committee on the Use of Animals at Hospital Sírio-Libanês (São Paulo, Brazil) under protocol number CEUA 2016/04.

### 4.3. Surgical Procedure for PD Model Induction

A rat model of PD was established as described previously [36,46,47]. Briefly, the animals were anesthetized with isoflurane (4% induction, 2.5% maintenance in 100% oxygen) associated with local anesthesia (2% lidocaine, 100 μL/animal on the scalp). Under stereotaxic conditions, 12 µg of 6-OHDA (Sigma-Aldrich, Burlington, MA, USA), a neurotoxin, diluted in 2 µL of 0.9% saline with 0.2% ascorbic acid, was injected at two different points into the left striatum (6 µg/µL of 6-OHDA at each point) [73]. The injection was performed using a Hamilton syringe at the following coordinates: +2.7 mm mediolateral, 0.0 mm anteroposterior and +4.5 mm dorsoventral (first point); +3.2 mm mediolateral, +0.5 mm anteroposterior and +4.5 mm dorsoventral (second point), according to the rat brain atlas [74]. Animals injected with 1 µL saline at two different points in the left striatum were used as controls. After injection, the needle was left in place for an additional 5 min to prevent backflow of the solution. After the striatal injection, animals were treated with non-steroidal anti-inflammatory drugs (NSAIDs) (0.5 mg/Kg, SQ, Meloxicam, Ourofino Pet, Cajamar, São Paulo, SP, Brazil), and penicillin/streptomycin as prophylactic antibiotics (0.2 mg/kg, intraperitoneal (i.p.); Zoetis, São Paulo, SP, Brazil). Saline and 6-OHDA with electrode implantation animals were returned to their home cages and monitored until complete recovery from anesthesia. The regular diet was supplemented with a dietary supplement (Ensure, Abbott, São Paulo, SP, Brazil) once daily for 3 consecutive days to ensure full recovery after nigrostriatal injury.

### 4.4. Surgical Procedure for Electrode Implantation

During the same surgical procedure, a set of 6-OHDA animals was implanted with insulated stainless-steel electrodes (250 μm in diameter with 0.55 mm of surface exposed, Plastic One, Roanoke, VA, USA) into the left STN, as previously described [36]. These cathode electrodes were implanted in the following coordinates: +2.5 mm mediolateral, -3.7 mm anteroposterior, and +7.5 mm dorsoventral [74]. Screws implanted on the skull over the parietal cortex (−6.0 anteroposterior and +2.5 lateral) were used as anodes. Electrodes and fixation screws were fixed to the skull using dental acrylic cement. After electrode implantation, animals were treated with NSAIDs and penicillin/streptomycin and received a dietary supplement, as previously described. The confirmation of electrode placement in the STN was performed retrospectively by analyzing Nissl-stained coronal sections in fixed brain sections obtained using a freezing sliding microtome, as well as in freshly frozen brain sections obtained using a cryostat, as previously described [36].

### 4.5. Surgical Procedure for Microdialysis Cannula Implantation

After the surgical procedure, 6-OHDA animals with or without STN electrodes were implanted with a stainless-steel guide cannula (14 mm length, 22 G) in the left striatum at the following coordinates: +2.7 mm mediolateral, 0.0 mm anteroposterior and +4.5 mm dorsoventral. The cannula was sealed to protect against obstruction and was only exposed for microdialysis collection. The cannula, with or without the electrode and fixation screws, was fixed to the skull using dental acrylic cement. After cannula implantation, animals were treated with NSAIDs and penicillin/streptomycin and received a dietary supplement.

### 4.6. Evaluation of Behavioral Immobility

To measure akinesia, we examined immobility using a bar (typical catalepsy test), during which the animal was placed with both forepaws on a 9 cm horizontal bar in an atypical posture, and the time necessary to correct the posture was recorded [75]. The behavioral immobility endpoint was considered when both forepaws were removed from the bar or when the animal moved its head in an exploratory manner.

### 4.7. Evaluation of the Apomorphine-Induced Rotational Behavior

To validate the PD model, animals were subjected to apomorphine-induced rotation seven days after the surgical procedure, as previously described [47]. Briefly, animals were injected with a dopaminergic agonist (apomorphine, 1 mg/kg, subcutaneous (s.c.), Tocris Bioscience, Ellisville, MI, USA) dissolved in 0.9% saline, and the number of rotations was recorded over 30 min using an automatic rotometer system (Rota-Count 8, Columbus Instruments, Columbus, OH, USA). No animal injected with 6-OHDA showed asymmetric rotational behavior.

### 4.8. DBS Protocol

Seven days after the surgical procedures, a group of 6-OHDA animals was treated with five sessions of DBS (6-OHDA + DBS ON–biphasic cathodic pulses at 130 Hz, 60 μs pulse width, 0.1 mA, 2 h/day) using a portable stimulator (St Jude MTS, St Jude Medical, Plano, TX, USA). DBS was applied for five days from 9:00 AM to 11:00 AM. To discriminate the effect of implant insertion, a group of animals underwent 6-OHDA injections and electrode implantation but received no stimulation (6-OHDA + DBS OFF).

### 4.9. Microdialysis Procedure

6-OHDA + DBS ON animals were subjected to intracerebral microdialysis before, during, and after the first and fifth subthalamic stimulation, corresponding to 8 and 12 days after striatal neurotoxin injection. In addition, 6-OHDA and 6-OHDA + DBS OFF animals were subjected to intracerebral microdialysis during the same period and under similar stimulation conditions. The procedures were performed according to a previously established protocol [76]. At the time of dialysate collection, the polyethylene tubes of the microdialysis probe were connected to a system of arms and switches, which allowed the connection of these tubes to a microinfusion syringe (CSF inlet) and microfraction collector (outlet of the dialyzed) refrigerated at 4 °C (Bicanalytical Systems, BAS, West Lafayette, IN, USA). In the experimental session, perfusion with ringer lactate (Baxter, sodium (Na^+^) 130.0 mEq/L, potassium (K^+^) 4.0 mEq/L, calcium (Ca^2+^) 3 mEq/L, chloride (Cl^−^) 109, 0 mEq/L, lactate (C_3_H_5_O_3_) 28 mEq/L, osmolarity: 272 mOsm/L, pH 6.0–7.5) was performed using an infusion pump, at a continuous flow of 2.0 µL/min. The sample collection was stabilized 90 min prior to baseline collection. Dialysate samples were collected every 20 min for up to 60 min after stimulation. After collection, the dialysate was stored at −80 °C until analysis. Striatal levels of glutamate and GABA were quantified using a high-performance liquid chromatography system (Shimadzu, UFLC Prominence) [77]. Six-OHDA and 6-OHDA + DBS OFF animals underwent collection for 120 min continuously, and the average of day 8 measurements was normalized by 100% and compared with the average measure obtained on day 12. For 6-OHDA + DBS ON animals, the average measured before stimulation on day 8 was normalized by 100% and compared with measurements during and after the first stimulation session (day 8), as well as before, during, and after the fifth stimulation session (day 12).

### 4.10. Immunohistochemistry

One hour after the last behavioral test (on day 13), selected animals were anesthetized with ketamine/xylazine (0.5/2.3 mg/kg, respectively, i.p.) and then subjected to transcardial perfusion with 0.9% saline solution, followed by 4% paraformaldehyde (PFA) dissolved in 0.1 M phosphate buffer (PB; pH 7.4) for immunohistochemistry assay. The assay was performed as described [46,47]. Briefly, brain slices were incubated with specific primary antibodies for mouse anti-tyrosine hydroxylase (TH) (1:1000; MAB5280, Millipore, Burlington, MA, USA), mouse anti-GFAP (1:1000, G3893, Sigma-Aldrich), rabbit anti-Iba-1 (1:1000; 019-19741, Wako, city, VA, USA), biotinylated secondary antibodies (1:200; Jackson ImmunoResearch, West Grove, PA, USA), and avidin-biotin-peroxidase complex (1:100; ABC Elite kit, Vector Labs, Burlingame, CA, USA), and visualized with diaminobenzidine tetrahydrochloride (DAB, Sigma-Aldrich). Images were captured using a light microscope (Eclipse E1000, Nikon, Manhattan, NY, USA). For NLRP3 immunofluorescence, striatal slices were incubated with a specific primary antibody for rabbit anti-NLRP3 (1:500, ab270449, Abcam, Burlington, MA, USA) and fluorescent secondary FITC antibody (1:500, Jackson ImmunoResearch, West Grove, PA, USA) and mounted with glycerol for immunofluorescence protection. Finally, images were captured using a fluorescence microscope (Eclipse E1000, Nikon). The region of interest, SN (from bregma −6.60 to −6.00 mm anteroposterior, −1.00 to −3.2 mm mediolateral, and −7.2 to −8.8 mm dorsoventral), and striatum (from bregma + 1.32 to + 0.12 mm anteroposterior, −2.00 to −5.00 mm mediolateral, and −4.00 to −8.00 mm dorsoventral) were identified using a stereotaxic atlas [74]. To establish the GFAP-IR and Iba-1-IR in the striatum, we evaluated five different sections (30 µm) per animal and from five animals per group. To quantify the sections by densitometry, we determined the threshold followed by the “analyze particles” plugin in the ImageJ software. Then, the average count determined by the software of the control rats (saline) was normalized to 100% to compare the percentage of modulation in the other groups.

### 4.11. Western Blotting

One hour after the last behavioral test (day 13), select animals were euthanized by decapitation, and the striatum was freshly dissected and gently homogenized at 4 °C in radioimmunoprecipitation assay (RIPA) buffer (50 mM Tris, 150 mM NaCl, 1 mM EDTA, 0.1% SDS, 0.5% deoxycholate, and 1% NP-40) with proteinase inhibitor cocktail (Thermo Fisher Scientific, Waltham, MA, USA). The protein concentration was measured using the Bradford protein assay (Bio-Rad, Hercules, CA, USA). The samples were diluted in Laemmli buffer for protein separation using sodium dodecyl sulfate-polyacrylamide gel electrophoresis (SDS-PAGE). Following electrophoretic separation, proteins were transferred to a nitrocellulose membrane (0.2 μm in diameter, ISEQ85R, Millipore), blocked for 1 h at room temperature with 5% bovine serum albumin in Tris-saline buffer with 0.1% Tween-20 (TBST), incubated overnight at 4 °C with rabbit anti-EAAT2 (1:5000, MAB2262, Millipore,) and mouse anti-β-actin (1:5000, #ab6046, Abcam), incubated for 2 h with the appropriate peroxidase-labeled secondary antibodies (1:2000, Jackson ImmunoResearch), developed using the chemiluminescence ECL Kit (Thermo Fisher Scientific), and analyzed to determine the density of the labeled bands using the ImageJ software (National Institutes of Health, Bethesda, MD, USA). Anti-β-actin was used as a loading control, and the control group (saline) was normalized to 100 for comparison with other groups.

### 4.12. Luminex

Striatal tissue samples, obtained and homogenized as previously described, were also analyzed by performing Luminex assays to quantify levels of chemokine/cytokine (chemokine (C-X3-C motif) ligand 1 (CX3CL1), tumor necrosis factor (TNF)-α, interleukin (IL)-1β, IL-4, IL-6, IL-10, IL-17a, and interferon (IFN)-γ; Millipore #RECYTMAG-65K), and for cell signaling (Akt, CREB, Erk, JNK, p65, p38, p70s6k, STAT3, and STAT5A; Millipore #48-680MAG). Analyses were performed according to the manufacturer’s recommendations.

### 4.13. Statistical Analysis

The animal sample size was established by considering cytokine expression as the primary outcome [78]. Results are expressed as the mean ± standard error of the mean (SEM). Data were analyzed using GraphPad Prism 9 (GraphPad Software, Inc., La Jolla, CA, USA). We performed two-way ANOVA (2-w-ANOVA) followed by Bonferroni’s multiple comparison post-hoc tests for apomorphine-induced rotation and glutamate and GABA concentrations during 6-OHDA and 6-OHDA + DBS OFF. In addition, we performed one-way ANOVA (1-w-ANOVA) followed by Tukey’s multiple comparison post-hoc test for inflammatory mediators, EAAT2, and p70s6k expression. Finally, for glutamate concentration in the 6-OHDA + DBS ON analysis, one-way repeated measures ANOVA (rm-1-w-ANOVA) was performed. In all cases, statistical significance was set at a *p*-value of < 0.05.

## 5. Conclusions

PD is a progressive disorder that induces chronic striatal inflammation and glutamatergic synaptic disturbances. Although STN-DBS can consistently improve PD symptoms in clinical practice despite the failure of pharmacological interventions, the mechanisms through which high-frequency stimulation or microlesion effects drive these improvements remain under investigation, especially regarding the striatal anti-inflammatory effect. Herein, we showed that although STN-microlesions display similar molecular mechanisms when compared with stimulated animals, STN-DBS can evoke a more precise control of glutamate clearance and more effectively reduces cell inflammation. Despite differences in experimental and clinical timeframes, the clinical benefits of STN-DBS, at least in the first weeks after electrode implantation, are probably related to the combined effect of focal microlesions and stimulation.

## Data Availability

The data that support the findings of this study are available from the corresponding author, upon reasonable request.

## Supplementary Materials

The following supporting information can be downloaded at: https://www.mdpi.com/article/10.3390/ijms232012116/s1.

## References

[1] Hornykiewicz O., Kish S.J. (1987). Biochemical pathophysiology of Parkinson’s disease. Adv. Neurol..

[2] McGeer P.L., McGeer P.L. (2007). Glial reactions in Parkinson’s disease. Mov. Disord..

[3] McGeer P.L., Yasojima K., McGeer E.G. (2001). Inflammation in Parkinson’s disease. Adv. Neurol..

[4] McGeer P.L., Itagaki S., Boyes B.E., McGeer E.G. (1988). Reactive microglia are positive for HLA-DR in the substantia nigra of Parkinson’s and Alzheimer’s disease brains. Neurology.

[5] Bartels A., Willemsen A., Doorduin J., de Vries E., Dierckx R., Leenders K. (2010). [11C]-PK11195 PET: Quantification of neuroinflammation and a monitor of anti-inflammatory treatment in Parkinson’s disease?. Park. Relat. Disord..

[6] Gerhard A., Pavese N., Hotton G., Turkheimer F., Es M., Hammers A., Eggert K., Oertel W., Banati R.B., Brooks D.J. (2006). In vivo imaging of microglial activation with [11C](R)-PK11195 PET in idiopathic Parkinson’s disease. Neurobiol. Dis..

[7] Kofler J., Wiley C.A. (2010). Microglia. Toxicol. Pathol..

[8] Saijo K., Winner B., Carson C.T., Collier J.G., Boyer L., Rosenfeld M.G., Gage F.H., Glass C.K. (2009). A Nurr1/CoREST Pathway in Microglia and Astrocytes Protects Dopaminergic Neurons from Inflammation-Induced Death. Cell.

[9] Ferrari C.C., Godoy M.C.P., Tarelli R., Chertoff M., Depino A.M., Pitossi F.J. (2006). Progressive neurodegeneration and motor disabilities induced by chronic expression of IL-1β in the substantia nigra. Neurobiol. Dis..

[10] McCoy M.K., Martinez T.N., Ruhn K.A., Szymkowski D., Smith C.G., Botterman B.R., Tansey K.E., Tansey M.G. (2006). Blocking Soluble Tumor Necrosis Factor Signaling with Dominant-Negative Tumor Necrosis Factor Inhibitor Attenuates Loss of Dopaminergic Neurons in Models of Parkinson’s Disease. J. Neurosci..

[11] Zhang W., Wang T., Pei Z., Miller D.S., Wu X., Block M.L., Wilson B., Zhang W., Zhou Y., Hong J.-S. (2005). Aggregated α-synuclein activates microglia: A process leading to disease progression in Parkinson’s disease. FASEB J..

[12] Pelvig D.P., Pakkenberg H., Stark A.K., Pakkenberg B. (2008). Neocortical glial cell numbers in human brains. Neurobiol. Aging.

[13] Liddelow S.A., Guttenplan K.A., Clarke L.E., Bennett F.C., Bohlen C.J., Schirmer L., Bennett M.L., Münch A.E., Chung W.-S., Peterson T.C. (2017). Neurotoxic Reactive Astrocytes Are Induced by Activated Microglia. Nature.

[14] Araque A., Parpura V., Sanzgiri R.P., Haydon P.G. (1999). Tripartite synapses: Glia, the unacknowledged partner. Trends Neurosci..

[15] Ventura R., Harris K. (1999). Three-Dimensional Relationships between Hippocampal Synapses and Astrocytes. J. Neurosci..

[16] Fenoy A.J., Goetz L., Chabardes S., Xia Y. (2014). Deep brain stimulation: Are astrocytes a key driver behind the scene?. CNS Neurosci. Ther..

[17] Newman E.A. (2003). New roles for astrocytes: Regulation of synaptic transmission. Trends Neurosci..

[18] Kimelberg H.K., Nedergaard M. (2010). Functions of astrocytes and their potential as therapeutic targets. Neurotherapeutics.

[19] Lehre K.P., Danbolt N.C. (1998). The Number of Glutamate Transporter Subtype Molecules at Glutamatergic Synapses: Chemical and Stereological Quantification in Young Adult Rat Brain. J. Neurosci..

[20] Eulenburg V., Gomeza J. (2010). Neurotransmitter transporters expressed in glial cells as regulators of synapse function. Brain Res. Rev..

[21] Anderson C.M., Swanson R.A. (2000). Astrocyte Glutamate Transport: Review of Properties, Regulation, and Physiological Functions. Glia.

[22] Rose C.R., Ziemens D., Untiet V., Fahlke C. (2018). Molecular and cellular physiology of sodium-dependent glutamate transporters. Brain Res. Bull..

[23] Rothstein J.D., Dykes-Hoberg M., Pardo C.A., Bristol L.A., Jin L., Kuncl R.W., Kanai Y., Hediger M.A., Wang Y., Schielke J.P. (1996). Knockout of Glutamate Transporters Reveals a Major Role for Astroglial Transport in Excitotoxicity and Clearance of Glutamate. Neuron.

[24] Chen Y., Vartiainen N.E., Ying W., Chan P.H., Koistinaho J., Swanson R.A. (2001). Astrocytes protect neurons from nitric oxide toxicity by a glutathione-dependent mechanism. J. Neurochem..

[25] Fahn S. (1999). Parkinson Disease, the Effect of Levodopa, and the ELLDOPA Trial. Arch. Neurol..

[26] Rascol O., Brooks D.J., Korczyn A.D., De Deyn P.P., Clarke C.E., Lang A.E. (2000). A Five-Year Study of the Incidence of Dyskinesia in Patients with Early Parkinson’s Disease Who Were Treated with Ropinirole or Levodopa. N. Engl. J. Med..

[27] Bezard E., Brotchie J., Gross C.E. (2001). Pathophysiology of levodopa-induced dyskinesia: Potential for new therapies. Nat. Rev. Neurosci..

[28] Benabid A.L. (2003). Deep brain stimulation for Parkinson’s disease. Curr. Opin. Neurobiol..

[29] Krack P., Batir A., Van Blercom N., Chabardes S., Fraix V., Ardouin C., Koudsie A., Limousin P.D., Benazzouz A., LeBas J.F. (2003). Five-Year Follow-up of Bilateral Stimulation of the Subthalamic Nucleus in Advanced Parkinson’s Disease. New Engl. J. Med..

[30] Kumar R., Lozano A., Kim Y.J., Hutchison W.D., Sime E., Halket E., Lang A. (1998). Double-blind evaluation of subthalamic nucleus deep brain stimulation in advanced Parkinson’s disease. Neurology.

[31] Benabid A.L., Chabardes S., Mitrofanis J., Pollak P. (2009). Deep brain stimulation of the subthalamic nucleus for the treatment of Parkinson’s disease. Lancet Neurol..

[32] Pereira J.L., Furie S., Sharim J., Yazdi D., DeSalles A.A., Pouratian N. (2016). Lateralization of the subthalamic nucleus with age in Parkinson’s disease. Basal Ganglia.

[33] Dafsari H.S., Reddy P., Herchenbach C., Wawro S., Petry-Schmelzer J.N., Visser-Vandewalle V., Rizos A., Silverdale M., Ashkan K., Samuel M. (2015). Beneficial Effects of Bilateral Subthalamic Stimulation on Non-Motor Symptoms in Parkinson’s Disease. Brain Stimul..

[34] Juri C., Oroz M.C.R., Obeso J.A. (2010). The pathophysiological basis of sensory disturbances in Parkinson’s disease. J. Neurol. Sci..

[35] Hamani C., Florence G., Heinsen H., Plantinga B.R., Temel Y., Uludag K., Alho E., Teixeira M.J., Amaro E., Fonoff E.T. (2017). Subthalamic Nucleus Deep Brain Stimulation: Basic Concepts and Novel Perspectives. eneuro.

[36] Campos A.C.P., Kikuchi D.S., Paschoa A.F.N., Kuroki M.A., Fonoff E.T., Hamani C., Pagano R.L., Hernandes M.S. (2020). Unraveling the Role of Astrocytes in Subthalamic Nucleus Deep Brain Stimulation in a Parkinson’s Disease Rat Model. Cell. Mol. Neurobiol..

[37] Iovino L., Tremblay M., Civiero L. (2020). Glutamate-induced excitotoxicity in Parkinson’s disease: The role of glial cells. J. Pharmacol. Sci..

[38] Ambrosi G., Cerri S., Blandini F. (2014). A further update on the role of excitotoxicity in the pathogenesis of Parkinson’s disease. J. Neural Transm..

[39] Sood A., Preeti K., Fernandes V., Khatri D.K., Singh S.B. (2021). Glia: A major player in glutamate–GABA dysregulation-mediated neurodegeneration. J. Neurosci. Res..

[40] Jech R., Mueller K., Urgošík D., Sieger T., Holiga Š., Růžička F., Dušek P., Havránková P., Vymazal J., Růžička E. (2012). The Subthalamic Microlesion Story in Parkinson’s Disease: Electrode Insertion-Related Motor Improvement with Relative Cortico-Subcortical Hypoactivation in fMRI. PLoS ONE.

[41] Holiga Š., Mueller K., Möller H.E., Urgošík D., Růžička E., Schroeter M.L., Jech R. (2015). Resting-state functional magnetic resonance imaging of the subthalamic microlesion and stimulation effects in Parkinson’s disease: Indications of a principal role of the brainstem. NeuroImage: Clin..

[42] Luo B., Lu Y., Qiu C., Dong W., Xue C., Zhang L., Liu W., Zhang W. (2021). Altered Spontaneous Neural Activity and Functional Connectivity in Parkinson’s Disease With Subthalamic Microlesion. Front. Neurosci..

[43] Alvarez L., Macias R., Lopez G., Pavon N., Rodriguez-Oroz M.C., Juncos J.L., Maragoto C., Guridi J., Litvan I., Tolosa E.S. (2005). Bilateral subthalamotomy in Parkinson’s disease: Initial and long-term response. Brain.

[44] Benazzouz A., Piallat B., Pollak P., Benabid A.-L. (1995). Responses of substantia nigra pars reticulata and globus pallidus complex to high frequency stimulation of the subthalamic nucleus in rats: Electrophysiological data. Neurosci. Lett..

[45] Benabid A., Pollak P., Gross C., Hoffmann D., Benazzouz A., Gao D., Laurent A., Gentil M., Perret J. (1994). Acute and Long-Term Effects of Subthalamic Nucleus Stimulation in Parkinson’s Disease. Ster. Funct. Neurosurg..

[46] Campos A.C.P., Berzuino M.B., Hernandes M.S., Fonoff E.T., Pagano R.L. (2019). Monoaminergic regulation of nociceptive circuitry in a Parkinson’s disease rat model. Exp. Neurol..

[47] Domenici R.A., Campos A.C.P., Maciel S.T., Berzuino M.B., Hernandes M.S., Fonoff E.T., Pagano R.L. (2019). Parkinson’s disease and pain: Modulation of nociceptive circuitry in a rat model of nigrostriatal lesion. Exp. Neurol..

[48] Higuchi Y., Matsuda S., Serizawa T. (2016). Gamma knife radiosurgery in movement disorders: Indications and limitations. Mov. Disord..

[49] Weintraub D., Elias W.J. (2016). The emerging role of transcranial magnetic resonance imaging-guided focused ultrasound in functional neurosurgery. Mov. Disord..

[50] Elias W.J., Lipsman N., Ondo W.G., Ghanouni P., Kim Y.G., Lee W., Schwartz M., Hynynen K., Lozano A.M., Shah B.B. (2016). A Randomized Trial of Focused Ultrasound Thalamotomy for Essential Tremor. N. Engl. J. Med..

[51] Hirsch E.C., Hunot S. (2009). Neuroinflammation in Parkinson’s disease: A target for neuroprotection?. Lancet Neurol..

[52] Pekny M., Nilsson M. (2005). Astrocyte activation and reactive gliosis. Glia.

[53] Freeman L., Guo H., David C.N., Brickey W.J., Jha S., Ting J.P.-Y. (2017). NLR members NLRC4 and NLRP3 mediate sterile inflammasome activation in microglia and astrocytes. J. Exp. Med..

[54] Chung E., Chen L., Chan Y., Yung K. (2008). Downregulation of glial glutamate transporters after dopamine denervation in the striatum of 6-hydroxydopamine-lesioned rats. J. Comp. Neurol..

[55] Meshul C., Emre N., Nakamura C., Allen C., Donohue M., Buckman J. (1998). Time-dependent changes in striatal glutamate synapses following a 6-hydroxydopamine lesion. Neuroscience.

[56] Robinson S., Freeman P., Moore C., Touchon J.C., Krentz L., Meshul C.K. (2003). Acute and subchronic MPTP administration differentially affects striatal glutamate synaptic function. Exp. Neurol..

[57] Amorim B.O., Covolan L., Ferreira E., Brito J.G., Nunes D.P., De Morais D.G., Nobrega J.N., Rodrigues A.M., DeAlmeida A.C.G., Hamani C. (2015). Deep brain stimulation induces antiapoptotic and anti-inflammatory effects in epileptic rats. J. Neuroinflammation.

[58] Wei L., Chen C., Ding L., Mo M., Zou J., Lu Z., Li H., Wu H., Dai Y., Xu P. (2019). Wnt1 Promotes EAAT2 Expression and Mediates the Protective Effects of Astrocytes on Dopaminergic Cells in Parkinson’s Disease. Neural Plast..

[59] Hardingham G.E., Bading H. (2010). Synaptic versus extrasynaptic NMDA receptor signalling: Implications for neurodegenerative disorders. Nat. Rev. Neurosci..

[60] Parsons M.P., Raymond L.A. (2014). Extrasynaptic NMDA Receptor Involvement in Central Nervous System Disorders. Neuron.

[61] Escartin C., Galea E., Lakatos A., O’Callaghan J.P., Petzold G.C., Serrano-Pozo A., Steinhäuser C., Volterra A., Carmignoto G., Agarwal A. (2021). Reactive astrocyte nomenclature, definitions, and future directions. Nat. Neurosci..

[62] Akwa Y., Di Malta C., Zallo F., Gondard E., Lunati A., Diaz-De-Grenu L.Z., Zampelli A., Boiret A., Santamaria S., Martinez-Preciado M. (2022). Stimulation of synaptic activity promotes TFEB-mediated clearance of pathological MAPT/Tau in cellular and mouse models of tauopathies. Autophagy.

[63] Kim K.I., Baek J.Y., Chung Y.C., Nam J.H., Shin W., Jin B.K. (2021). p70S6K on astrocytes protects dopamine neurons from 1-methyl-4-phenylpyridinium neurotoxicity. Glia.

[64] Herrington T.M., Cheng J.J., Eskandar E.N. (2016). Mechanisms of deep brain stimulation. J. Neurophysiol..

[65] Yang J., Hu L.-F., Liu X., Zhou F., Ding J.-H., Hu G. (2006). Effects of iptakalim on extracellular glutamate and dopamine levels in the striatum of unilateral 6-hydroxydopamine-lesioned rats: A microdialysis study. Life Sci..

[66] Centonze D., Gubellini P., Rossi S., Picconi B., Pisani A., Bernardi G., Calabresi P., Baunez C. (2005). Subthalamic nucleus lesion reverses motor abnormalities and striatal glutamatergic overactivity in experimental parkinsonism. Neuroscience.

[67] Mallet N., Ballion B., Le Moine C., Gonon F. (2006). Cortical Inputs and GABA Interneurons Imbalance Projection Neurons in the Striatum of Parkinsonian Rats. J. Neurosci..

[68] Tritsch N.X., Ding J.B., Sabatini B.L. (2012). Dopaminergic neurons inhibit striatal output through non-canonical release of GABA. Nature.

[69] Obeso J.A., Rodriguez-Oroz M.C., Rodriguez M., Lanciego J.L., Artieda J., Gonzalo N., Olanow C.W. (2000). Pathophysiology of the basal ganglia in Parkinson’s disease. Trends Neurosci..

[70] Tawfik V.L., Chang S.-Y., Hitti F.L., Roberts D.W., Leiter J.C., Jovanovic S., Lee K.H. (2010). Deep Brain Stimulation Results in Local Glutamate and Adenosine Release. Neurosurgery.

[71] Lee K.J., Shim I., Sung J.H., Hong J.T., Kim I.S., Cho C.B. (2017). Striatal Glutamate and GABA after High Frequency Subthalamic Stimulation in Parkinsonian Rat. J. Korean Neurosurg. Soc..

[72] Dvorzhak A., Gertler C., Harnack D., Grantyn R. (2013). High Frequency Stimulation of the Subthalamic Nucleus Leads to Presynaptic GABA(B)-Dependent Depression of Subthalamo-Nigral Afferents. PLoS ONE.

[73] Chudler E.H., Lu Y. (2008). Nociceptive behavioral responses to chemical, thermal and mechanical stimulation after unilateral, intrastriatal administration of 6-hydroxydopamine. Brain Res..

[74] Paxinos G., Watson C. (2005). The Rat Brain in Stereotaxic Coordinates.

[75] Sanberg P.R. (1980). Haloperidol-induced catalepsy is mediated by postsynaptic dopamine receptors. Nature.

[76] de Andrade E.M., Martinez R.C.R., Pagano R.L., Lopes P.S.S., Auada A.V.V., Gouveia F.V., Antunes G.F., Assis D.V., Lebrun I., Fonoff E.T. (2020). Neurochemical effects of motor cortex stimulation in the periaqueductal gray during neuropathic pain. J. Neurosurg..

[77] Martinez R.C., Hamani C., De Carvalho M.C., De Oliveira A.R., Alho E., Navarro J., Ghilardi M.G.D.S., Bor-Seng-Shu E., Heinsen H., Otoch J.P. (2013). Intraoperative dopamine release during globus pallidus internus stimulation in Parkinson’s disease. Mov. Disord..

[78] Charan J., Kantharia N.D. (2013). How to calculate sample size in animal studies?. J. Pharmacol. Pharmacother..

