# Supplementary material for: Effect of Subthalamic Stimulation and Electrode Implantation in the Striatal Microenvironment in a Parkinson’s Disease Rat Model
Source: Int J Mol Sci. 2022 Oct 11;23(20):12116. doi: 10.3390/ijms232012116 (PMC9603133; doi:10.3390/ijms232012116)
Supplement: Supplementary file 1 [file ijms-23-12116-s001.zip › ijms-1852789-supplementary.pdf]

International Journal of *Molecular Sciences*

Supplementary materials

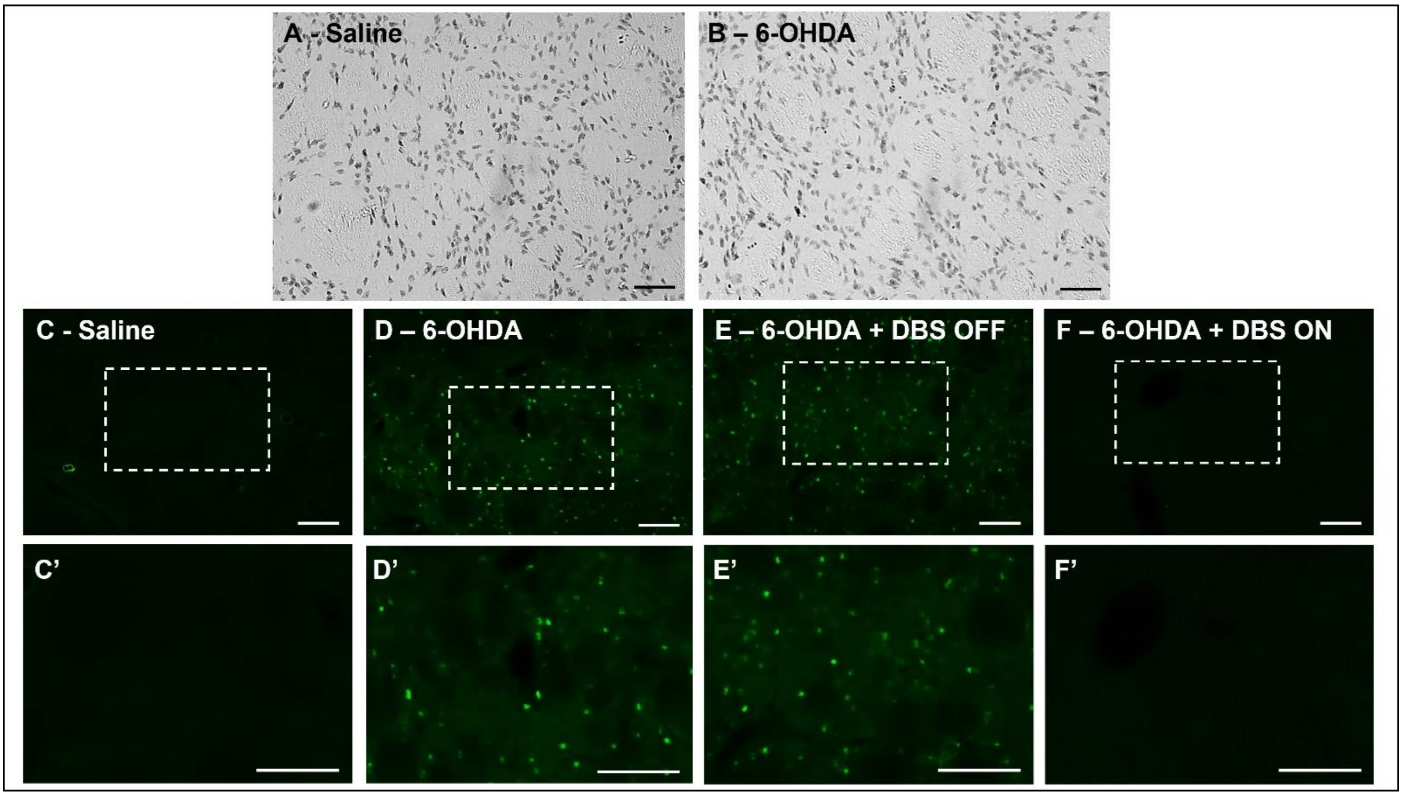

Figure S1. Representative photomicrographs of IR-NeuN in the striatum of saline (**A**) and 6-OHDA (**B**) rats. Representative photomicrographs of IR-NLRP3 in the striatum of saline (**C**, C′), 6-OHDA (**D**, D′), 6-OHDA + DBS OFF (**E**, E′) and 6-OHDA + DBS ON (**F**, F′). Scale bar: 100 μm.
